# Left Atrial Appendage Occlusion Guided Only by Transesophageal Echocardiography

**DOI:** 10.1155/2019/1376515

**Published:** 2019-01-02

**Authors:** Jinlong Zhao, Feng Li, Yueli Zhang, Zhongyun Zhuang, Man Wang, Liang Fu, Yinkai Ni, Zhexin Lu, Zonghui Chen, Cheng Zhang

**Affiliations:** ^1^Department of Cardiovascular Surgery, Shanghai Jiao Tong University Affiliated Sixth People's Hospital, Shanghai, China; ^2^Department of Ultrasound Medicine, Shanghai Jiao Tong University Affiliated Sixth People's Hospital, Shanghai, China; ^3^Shanghai Push Medical Device Technology Inc, Shanghai, China

## Abstract

**Aims:**

To investigate a new method of left atrial appendage occlusion without fluoroscopy.

**Methods and Results:**

We performed left atrial appendage occlusion for 14 patients with atrial fibrillation in our hospital. All of the surgeries were completed in a general surgery setting, avoiding fluoroscopy, and in each case, the entire procedure was guided by transesophageal echocardiography (TEE). All of the surgeries were performed through the femoral vein pathway. All operations went smoothly with no serious complications. Postoperative TEE indicated that each device was in a good position, and there was no residual shunt around any of the devices.

**Conclusions:**

TEE-guided left atrial appendage occlusion is safe and reliable, simplifies the procedure, protects doctors and patients from radiation, and is gradually becoming the mainstream operation for left atrial appendage occlusion. This trial is registered with ChiCTR1800018387.

## 1. Introduction

Atrial fibrillation is the most common type of persistent arrhythmia. In addition to causing palpitations and discomfort, the onset of atrial fibrillation increases the risk of thromboembolism [1]. Evidence reported in the literature both at home and abroad indicates that thrombosis from the left atrial appendage is the main cause of stroke in atrial fibrillation [2]. Percutaneous left atrial appendage occlusion (LAAO), a new technique for preventing thromboembolism, offers more options for patients with atrial fibrillation who are at increased risk of blood clotting and cannot take anticoagulants. Currently, the clinical application of LAAO technology is guided by fluoroscopy under general anesthesia. The technology has a long learning curve, increases radiation risk, and is expensive. Moreover, the implantation procedure can cause serious complications [3–6]. Therefore, we chose to perform LAAO under the guidance of transesophageal echocardiography (TEE) in a general surgery operating theater and truly achieved zero exposure to radiation. The operating times were short, and there were fewer complications compared with the fluoroscopy-guided procedure. This technology should be available in the clinic as it is safe, radiation-free, and effective.

## 2. Methods

Written informed consent was obtained from each patient. Procedures were performed in accordance with ethical standards. From March 2018 to November 2018, percutaneous LAAO was performed with the guidance of TEE in a total of 14 patients. All patients underwent LAA closure under general anesthesia.

### 2.1. Inclusion/Exclusion Criteria

The CHA2DS2-VASC scores of the patients included in this group were greater than or equal to 2. Furthermore, these patients were not suitable for long-term oral anticoagulation, and stroke or embolism events could still occur on the basis of the INR standard after taking warfarin.

Exclusion criteria were inner diameter of LA > 65 mm, spontaneous development of intracardiac thrombus, severe mitral valve lesions and pericardial effusion > 3 mm, life expectancy <1 year, low risk of stroke or low risk of bleeding, taking warfarin for other reasons, and complex atherosclerotic plaques in the ascending aorta/aortic arch. Patients undergoing elective cardiac surgery whose patent foramen ovale (PFO) was not closed with an atrial septal aneurysm and right-left shunt and who were in cardiac dysfunction were also excluded.

### 2.2. Occluder Device

We choose the LAmbre™ occluder device (Lifetech) for occlusions by the femoral vein pathway because it can be applied to a variety of left appendage structures and has a unique hook anchoring design that creates a more stable block.

### 2.3. Device Implantation

**Figure 1:**
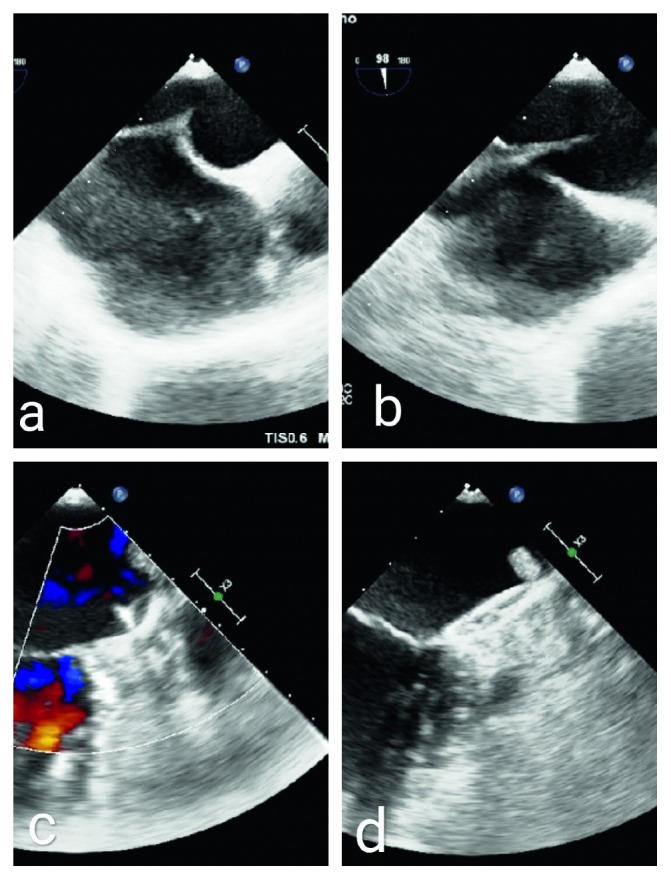
Operation procedure of percutaneous occlusion of the left atrial appendage through the femoral vein pathway. (a) The tent structure. (b). Successful atrial septum puncture. (c) Push-and-pull test. (d) Closure with successful release.

The procedure for percutaneous occlusion of the left appendage through the femoral vein pathway was as follows (Figure 1). After successfully puncturing the right femoral vein, the head end of the soft guidewire was implanted into the superior vena cava under the guidance of TEE. The transseptal guiding introducer pushed the atrial septum to the left side of the atrial septum under the guidance of TEE, creating a structure similar to a “tent.” After the transseptal needle successfully punctured the atrial septum, the puncture sheath was transported to the left atrium. Then, the Amplatzer super stiff guidewire was inserted, and its head end was placed in the upper left pulmonary vein under the guidance of TEE. The transseptal guiding introducer was replaced by the delivery sheath, and the head end of the sheath was placed on the left atrium, and then, the Amplatzer super stiff guidewire was replaced with a pigtail catheter. Under the guidance of TEE and the pigtail catheter, the head end of the delivery sheath was transported to the left appendage. The left appendage occlusion device, which was measured in advance and well vented, was transported to the LAA through the sheaths with close TEE monitoring, and the push-and-pull test showed that there was almost no residual blood in the left appendage. When the occluder was in a good position, the cable and sheath were removed.

### 2.4. End Points

The end points of this study refer to the standardized end points/criteria included in the Munich consensus paper on LAAO by Tzikas et al. [7]. The primary end point of the study included successful implantation of the LAA occlusion device. Successful closure of the LAA was determined by TEE as the absence of flow or minimal flow (jet of <5 mm width; we set it to be < 3 mm width) around the device according to the echocardiographic sealing criteria was described previously [8]. The second end points were the occurrence of adverse events, which included composite end points such as all-cause mortality, ischemic stroke or systemic embolism, and periprocedural complications including pericardial effusion/tamponade, bleeding, pericarditis, myocardial infarction, access-related complications, renal and hepatic injuries, and device-related complications.

### 2.5. Statistical Analysis

SPSS 22.0 software was used for the statistical analysis. Estimated frequencies of event occurrences are expressed as percentages or rates. Continuous variables are summarized as the mean and SD.

## 3. Results

### 3.1. Baseline Characteristics

The average age of patients in this group, including 9 males and 5 females, was 67.1 ± 9.5 years, and all of them had atrial fibrillation. Nine patients had hypertension before the operation, 4 patients had a history of cerebral infarction, one of the patients had diabetes, and none of the patients had valve disease. The average CHA2DS2VASc score was 3.5 ± 2.1, and the average HASBLED score was 3.6 ± 0.9 (Table 1).

### 3.2. Primary End Points

TEE-guided LAAO was successfully completed for all patients in the group. The mean LAA width was 23.9 ± 1.9 mm (Table 2), and the mean LAA work length was 22.8 ± 7.2 mm. The mean diameter of the seal plate of the devices was 27.7 ± 3.3 mm. All of the devices were implanted successfully using the first choice with no changes to other types of occluders. Because some of the procedures were combined with radiofrequency ablation of atrial fibrillation, the plugging time alone could not be calculated accurately. Simple percutaneous closure of the LAA without radiofrequency ablation of atrial fibrillation could be completed within 30 minutes.

### 3.3. Secondary End Point

No complications that seriously affected a patient's life occurred during the perioperative period. During the operation, there was one patient who developed a reaction to the anesthesia (nausea and vomiting after surgery) (Table 3). The endotracheal tube was removed immediately after the completion of surgery. Postoperative TEE reexamination showed successful closure of the LAA in all patients with no flow or minimal residual flow of 2 mm in one patient. The mean postoperative hospitalization time was 4.5 ± 1.3 days.

## 4. Discussion

Patients with atrial fibrillation (AF), which is the most common arrhythmia, have an increased risk for stroke [1] ranging from 2% to >10% per year, depending on additional risk factors [9]. As a result, AF is responsible for 15% to 20% of all ischemic strokes [10].

The mortality and disability rates of atrial fibrillation are high, which seriously threaten the life and quality of life of patients. Warfarin anticoagulation is the first choice of therapy, but due to the existence of anticoagulant contraindications and other factors, some patients refuse drug treatment or are not allowed for drug treatment. According to the statistics, more than 90% of thrombi of patients with nonvalvular atrial fibrillation originate in the left atrial appendage. In recent years, many clinical studies in home and abroad have shown that LAAO can reduce the risk of stroke in patients with atrial fibrillation. A multicenter clinical study of 110 patients showed that treatment with LAAO can reduce the risk of stroke, major bleeding, and death compared with other therapeutic strategies [11].

The European Society of Cardiology Guidelines for managing atrial fibrillation stated in 2016 that LAA occlusion may be considered for stroke prevention in patients with AF and contraindications for long-term anticoagulant treatment (e.g., those with a previous life-threatening bleed without a reversible cause). Class IIb, level B surgical occlusion or exclusion of the LAA may be considered for stroke prevention in patients undergoing thoracoscopic AF surgery. Class IIb, level B surgical LAA occlusion or exclusion concomitant to cardiac surgery has been performed for many decades and with various techniques. Multiple observational studies have indicated the feasibility and safety of surgical LAA occlusion/exclusion, but only limited controlled trial data are available [12–15]. Residual LAA flow or incomplete LAA exclusion can increase stroke risk [16].

Currently, the most commonly used technique in clinical practice is LAAO via percutaneous transcutaneous catheter under the guidance of angiography and TEE. Such an operation process is complex, the operating time is long, and the incidence of complications is relatively high; rates for major complications, including significant pericardial effusion, tamponade, and periprocedural stroke, are reported to be between 3.7% and 7.7% [2, 17]. More importantly, it increases the exposure time of doctors and patients to X-rays. According to the relevant literature and our accumulated experience in the occlusion of congenital heart disease induced by pure esophageal ultrasound, we believe that the above procedures can be completely replaced by the 2D and 3D functions of TEE, which can demonstrate the shape of the LAA, the diameter of the LAA inlet and waist, and the location of the occlusion device. Radinovic et al. [18] reported the technique of atrial septal puncture guided by pure esophageal ultrasound but did not carry out further left heart operations under pure esophageal ultrasound guidance. Since the beginning of this year, our center has successively developed percutaneous catheter LAAO technology that is completely guided by TEE, avoiding exposure to radiation, shortening the operating time greatly, and reducing perioperative complications.

The regular 2D transesophageal echocardiogram can show each side of the LAA, enabling observation of the presence of a thrombus and measurement of its largest and least diameters and the depth of the LAA. However, there is no imaging advantage for an LAA with a complex structure or different opening forms.

Three-dimensional TEE can be used to quickly obtain a perpendicular LAA section, and the multisection surface can display the diameter of the LAA opening in real time, reduce the steps required during surgery, and shorten the measurement time. It can directly image the complex anatomical structure of the LAA and display its shape, internal structure, and thrombus [19] in 3D images. Therefore, it plays an important role in screening patients, selecting a suitable plugging device, and ensuring the sealing effect.

After the successful release of the plugging device, TEE can evaluate its position and residual shunt at multiple angles and on multiple planes. More importantly, TEE can dynamically display the changes in the above observation indexes during the pushing and pulling experiment in real time.

To summarize, the successful completion of percutaneous transcatheter LAAO, purely TEE-guided atrial septal puncture, and the release of the occluder is the key to the success of this procedure. Since many patients in atrial fibrillation have a right atrium enlargement, and the inferior vena cava and atrial septum are not completely in the same plane, and the normal atrial septum puncture catheter may not succeed in moving the atrial septum to the left atrial surface after it is ultrasonically guided to the atrial septum position, so that the puncture may cause the heart to rupture. We used a modified atrial septal puncture catheter, which has a larger angle and a longer length from the angle to the tip of the catheter. Under the protection of a guidewire and the guidance of TEE, it is easy to create a “tent” structure at the atrial septum. We still choose to puncture the lower part of the atrial septum, so that the guidewire and catheter can enter the LAA more easily. 2D and 3D TEE can monitor the puncture process in real time to ensure the safety of the procedure. Secondly, before releasing the occluder, the conveying sheath should be placed in the upper left pulmonary vein, and it is afterwards guided by a pigtail catheter to the left atrial appendage; this prevents penetration of bleeding from the left atrial appendage. In one patient, the postoperative X-ray suggested a high density shadow in the upper left lungs, considered to be upper left pulmonary vein branch bleeding, but if it were LAA bleeding, it could lead to serious cardiac tamponade.

Berti et al. reported intracardiac echocardiography- (ICE-) guided LAAO [20]. According to their experience, ICE represents a safe and useful ultrasound option for guiding the LAA transcatheter occlusion procedure and for preventing short- and midterm complications. It has the advantage over TEE of not requiring the support of general anesthesia and anesthesiology. But crucially, it still requires fluoroscopy. With the wide applications of ICE, we have reasons to believe that LAAO by the femoral vein pathway can be carried out under local anesthesia with the guidance of pure ICE, with minimal injury to patients in a minimally invasive and green procedure.

### 4.1. Study Limitations

There were several limitations in our present study. This single-center study was small and nonrandomized. There was no control group. Further study will analyze LAAO guided by pure TEE and fluoroscopy and compare the two methods.

## 5. Conclusions

LAAO is feasible and safe under the guidance of TEE.

### 5.1. Impact on Daily Practice

TEE-guided LAAO is safe and reliable, and the role of TEE is most likely to be further explored.

## Figures and Tables

**Table 1:** Baseline patient characteristics.

	*n*=14
Age (y)	67.1 ± 9.5
Sex (male/female)	9 (64.3%)/5 (35.7%)
Congestive heart failure/LV dysfunction	5 (35.7%)
Hypertension	9 (64.3%)
Diabetes mellitus	1 (7.1%)
Stroke/TIA/TE	4 (28.6%)
Vascular disease	5 (35.7%)
CHA2DS2VASc score, *n*	
0 to 1	0
2	7
3	2
4	1
5	0
6	1
7	1
8	1
Mean CHA2DS2VASc score	3.5 ± 2.1
Mean HASBLED score	3.6 ± 0.9

LV, left ventricular; TIA, transient cerebral ischemic attacks; TE, thromboembolism.

**Table 2:** Procedure findings.

	*n*=14
TEE assessment	
LAA ostium width (mm)	23.9 ± 1.9
LAA work length (mm)	22.8 ± 7.2
Diameter of seal plate of the devices (mm)	27.7 ± 3.3
Number of devices, *n*	1

LAA, left atrial appendage; TEE, transesophageal echocardiography.

**Table 3:** Perioperative complications.

	*n*=14
Major complications	0 (0%)
Cardiac tamponade	0
Stroke	0
Myocardial infarction	0
Device dislocation	0
Malignant arrhythmia	0
Death	0
Minor complications	2 (14.3%)
Pericardial effusion	0
Residual shunt	0
Thrombus	0
Access-related complications/hematoma	1
Anesthesia reaction	1
Pericarditis	0
Renal and hepatic injuries	0
Device-related complications	0
All complications	2 (14.3%)

## Data Availability

The data used to support the findings of this study are included within the tables of the article.
